# Research and Validation of CF/PEEK-Based Truss Rod Crimping and Pultruding Process for On-Orbit Isoform Forming

**DOI:** 10.3390/ma17102393

**Published:** 2024-05-16

**Authors:** Jiayong Yan, Peng Li, Chao Geng, Xuanyu Guo, Lixin Zhang

**Affiliations:** 1 Mechanical Engineering College, Yanshan University, Qinhuangdao 066004, China; yanjiayong0713@126.com (J.Y.);; 2 Beijing Spacecraft Manufacturing Co., Ltd., Beijing 100094, China; 13910506063@163.com; 3 Hebei Innovation Center for Equipment Lightweight Design and Manufacturing, Qinhuangdao 066004, China

**Keywords:** on-orbit iso-material construction, crimping and pultrusion forming, CF/PEEK

## Abstract

A crimping and pultruding forming process for truss rods using Carbon Fiber (CF)/Polyether–Ether–Ketone (PEEK) prepreg tape as the raw material is proposed to address the problem of continuous manufacturing of space trusses on orbit. The proposed process provides material rods for continuous truss manufacturing. Through numerical simulation and experimental verification, the effects of relevant parameters on the forming process are determined, an efficient method of rod curl pultrusion, in-rail, equal material forming is proposed, and the structural configuration of the rod curl pultrusion forming mold is determined. The equivalent macroscopic mechanical properties of unidirectional CF/PEEK prepreg strips are considered, and the rod-forming process is investigated. Rod samples with different process parameters are prepared, and several tests are conducted on them. The results show that the forming load pull is negatively correlated with the temperature at the same forming speed, and forming speed is positively correlated with the forming load pull at a certain temperature. Temperature and speed affect the surface quality of the rod, the density of the material filling, and the mechanical properties of the rod. The optimal forming process parameters are determined through numerical simulation and experimental verification. The developed molding technology has the advantages of high efficiency, low energy consumption, and high integration. It reduces manufacturing costs and improves manufacturing efficiency, so it can serve as a new and effective solution for the manufacturing of high-performance truss rods in the aerospace field.

## 1. Introduction

With the deepening of human exploration and space utilization, traditional space missions now require the unfolding of the entire space structure after being launched into a predetermined orbit in one go, which leads to a huge launch system and high costs. Large-scale structures, such as megawatt-class solar power plants and space radio telescopes, can be rapidly constructed using raw material replenishment and on-orbit fabrication and assembly [1], thus overcoming the limitations in rocket size at the scale of truss structures.

The rod material that makes up the truss structure is formed by the integrated forming of composite strip crimping and pultruding, which can realize the efficient and rapid forming of slender, continuous, high-stiffness rods under low power consumption. Compared with metal rods, composite rods with matrix materials have a low melting point, require a low temperature for the forming process, and have low tensile force, so the power consumption and volume of the equipment are comparatively small. Composite rods are lightweight and have high strength and corrosion resistance, so they can adapt to the special environment of space [2].

Compared with subtractive and additive manufacturing, iso-material manufacturing yields rods with high strength and high precision with a continuous length, thus ensuring consistency between the structural performance and assembly precision of the constituent trusses [3,4]. Moreover, the forming process is reliable, efficient, and fast, which reduces problems and shortens the fabrication cycle. The forming molds can also be easily repaired and replaced, thus prolonging the service life. In summary, iso-material manufacturing is suitable for forming rods for the special on-orbit manufacturing environment [5].

In the Trusselator Project of Tethers Unlimited Inc., carbon fiber prepreg tape was used as the raw material to fabricate rods by pultrusion molding; the rods were connected to form a truss structure. The cross-section of the truss samples was formed of equilateral triangles with side lengths of 75 mm, and the truss was used to mount solar panels [6,7]. The Archinaut Project successfully achieved additive manufacturing by using extended structural additive manufacturing machine technology in a thermal-vacuum, space-like environment, and a 37.7 m trussed girder structure was fabricated [8]. San Diego Composites designed an advanced continuous composite truss printing system to fabricate lightweight composite trusses for solar array support structures in situ after launch [9].

Professor Jiang Shengyuan’s team from the Harbin Institute of Technology proposed a continuous on-orbit truss construction method based on strip connection forming [10]. The method relies on the principle of using longitudinal, transverse, and diagonal beams generated from the strip material to weave continuous truss structures under the longitudinal beams’ traction and the transverse and diagonal beams’ entanglement. The fast, efficient forming of truss structures by using composite materials is a future trend in the fabrication of large space structures on orbit.

With the progress in manufacturing, extrusion forming technology is playing an increasingly important role in the field of composite materials. As a highly automated manufacturing process, extrusion molding is directly affected by the pulling speed, and therefore has attracted much attention. Vedernikov, Alexander N.’s research found that different extrusion speeds have a significant impact on the structural characteristics and mechanical properties of extruded glass fiber/epoxy vinyl resin composite products [11]. There are significant differences in the mechanical properties between products produced at low speeds and those produced at high speeds, mainly manifested in matrix cracking and other aspects. At high speeds, products are prone to aggregation and delamination, resulting in changes in their strength and Young’s modulus, as well as a decrease in their interlayer shear strength.

In addition, Vedernikov, Alexander N. also investigated the relationship between traction speed and flat laminated sheets of glass fiber/vinyl ester resin structural composite materials in the extrusion molding process [12]. The experimental results show that as the traction speed increases, the mechanical characteristics of the product change, accompanied by an increase in bubbles, longitudinal voids, and matrix cracks, as well as an increase in density and size. This study confirms for the first time the feasibility of high-speed extrusion forming for producing large-section profiles suitable for structural applications.

On the other hand, Tucci, F.’s research focuses on the extrusion molding of pre-impregnated glass-fiber-reinforced polypropylene strips [13]. By analyzing the experimental results, they gained a deeper understanding of the properties of the material–mechanical interaction and the changes in the polypropylene matrix. The results indicate that the extruded products have good quality and mechanical properties, suitable for engineering applications, but further research and optimization are still needed to improve the uniformity and performance stability of the products.

In this study, in consideration of adaptability to the on-orbit manufacturing environment, a CF/PEEK-based truss rod crimping and pultrusion iso-material forming process is proposed to address the need for longitudinal rod forming and continuous winding of diagonal rods in truss structures. The load and PEEK phase transition of the rod-forming process are numerically simulated, and the influence mechanism of the rod crimping and pultrusion forming process is experimentally verified using different process parameters. In addition, the optimal forming process parameters are determined. This work provides efficient, rapid rod-forming methods and process guidance for the continuous manufacturing of truss structures on orbit. Using the proposed process, the design of the truss winding and forming scheme and the performance of the truss structure can be determined.

## 2. Principle of Equal Material Forming for Truss Rods

### 2.1. Truss Continuous Forming Manufacturing Options

**Figure 1:**
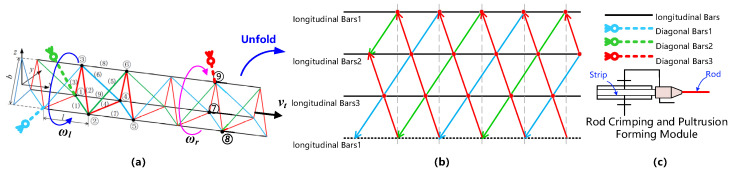
Principal diagram of rod-based truss winding forming. (**a**) Truss winding method based on rod; (**b**) unfolded diagram of continuous winding forming of truss; (**c**) schematic diagram of rod forming method.

The manufacturing forms of truss structures are mainly classified into rod parts assembly, additive manufacturing, and winding forming. Considering the high efficiency, rapidity, and forming accuracy of truss manufacturing, this study investigates the crimping and pultruding forming process of basic material rods for truss structures via winding. As presented in Figure 1a, the triangular truss structure formed by winding consists of longitudinal and diagonal bars. The diagonal bars with the same color in the unfolding diagram in Figure 1b are arranged in the same vertical direction, indicating that each diagonal bar is continuous. The longitudinal bars should be continuous as well to satisfy the requirements of the truss length. Therefore, the truss rods are formed from continuous strips via the winding and pultruding forming process to create continuous rods with certain cross-sectional shapes, as shown in Figure 1c. The winding process of the diagonal rods utilizes rods that are not completely cooled and cured in the pultrusion forming process and thus have a certain flexibility; they are continuously wound around the longitudinal rods in accordance with the specific winding procedure. Then, the truss structure is formed through the connection of the nodes.

### 2.2. Principle of Crimping and Pultruding of Truss Rods

**Figure 2:**
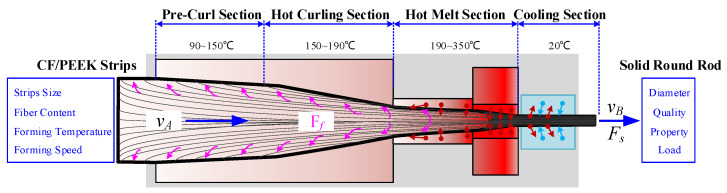
Principle of rod curling and pultrusion forming. The red arrow represents heat flow, and the blue arrow represents cold flow.

As shown in Figure 2, first, the CF/PEEK unidirectional prepreg tape enters the precurling section of the forming mold. The strip in a highly elastic state of viscoelasticity is used to achieve the precurl, and its stiffness is increased when it is pushed forward to avoid the frictional resistance caused by the inner wall of the cavity. This frictional resistance causes the wrinkles to form in the strip, which clog the mold. Second, the precurl of the strip enters the hot-curl section, in which the diameter of the hollow tube formed by the curling is gradually reduced, and the push force is maintained to realize gradual extrusion. After entering the hot-melt section, the base PEEK material enters in a viscous flow state at high temperature. The movement of the molecular chain of PEEK becomes increasingly intense and exhibits high mobility and plasticity under the actions of extrusion and high temperature. PEEK and carbon fiber filaments are produced in the structure of the hot-melt forming molds under the constraints of the formed solid cross section of the rod. Lastly, the cooling module converts PEEK from a viscous flow state to a glassy state, completes the cooling and shaping of the rod, and carries out the continuous conveying of the rod through the traction mechanism. The input parameters, such as strip size, fiber content, forming temperature, and speed, during the rod forming process affect the dimensional accuracy, surface quality, mechanical properties, and forming haul-off loads of the formed rods.

**Figure 3:**
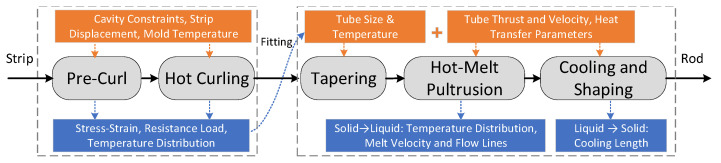
Process path of rod curling and pultrusion molding.

The forming process from strip to rod involves precurling, hot curling, tapering, hot-melt pultrusion, cooling, and shaping, as shown in Figure 3. Precurling and hot material’s curling realize the transition from a glassy strip to a highly elastic tubing, and the result of the process is used as an input for the next step. After tapering, hot-melt pultrusion, cooling, and shaping, the base PEEK material undergoes a highly elastic–viscous–fluidic–glassy transition to generate a solid rod.

## 3. Numerical Simulation of Rod-Forming Process

### 3.1. Temperature Distribution of Forming Molds

**Figure 4:**
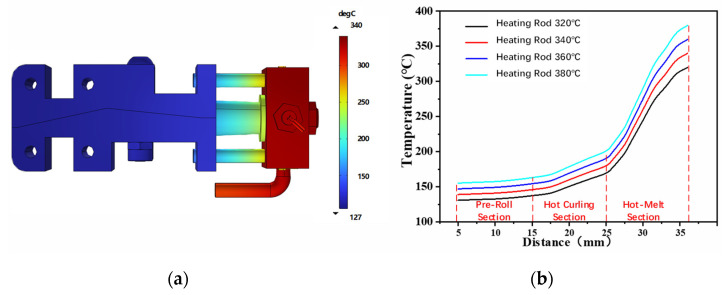
Forming mold temperature simulation results. (**a**) Under the temperature setting of 340 °C for the heating rod; (**b**) temperature gradient distribution of heating rods under different temperature conditions.

In accordance with the requirements of the temperature gradient of each interval segment in the principle of rod crimping and pultrusion forming, thermal simulation design of the tube structure size of the forming die was carried out to ensure the heat transfer balance from the heat source to the crimping die, and a heat insulation structure is added to the part of the crimping die that is solidly connected to the conduit to prevent excessive heat transfer. Figure 4a shows the temperature distribution under the condition that the temperature of the heating rod is 340 °C. The temperature of the curling mold is slightly higher than the glass transition temperature of PEEK at 143 °V, which softens the strip during the curling process and reduces the load generated by the curling deformation when the strip enters the mold. The temperature of the conduit is lower than the melting point of the PEEK to prevent the PEEK material from melting prematurely and producing wrinkles, which would result in mold clogging. Figure 4b shows the temperature profiles of the precurling, hot-curling, and hot-melt sections with the heating rod set to several temperatures. The profiles are used as the temperature boundary conditions in the subsequent curl-forming simulation process.

### 3.2. Tube Curling and Forming

After the CF/PEEK unidirectional prepreg tape enters the crimping mold, it undergoes elastic–plastic deformation to form the tubing under the constraint of the mold cavity and mold temperature. During this process, the forming speed and mold temperature affect the forming load pull of the tubing, and the influencing law is investigated through numerical simulation. For the composite material properties of the CF/PEEK unidirectional prepreg strips, the equivalence of their macroscopic mechanical properties is obtained by selecting a representative volume unit (RVE) for their fine structure and by homogenizing them using periodic boundary conditions [14,15].

**Figure 5:**
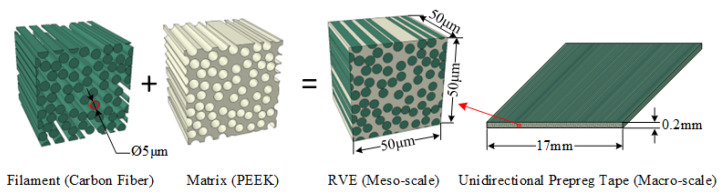
CF/PEEK unidirectional prepreg tape composition.

**Figure 6:**
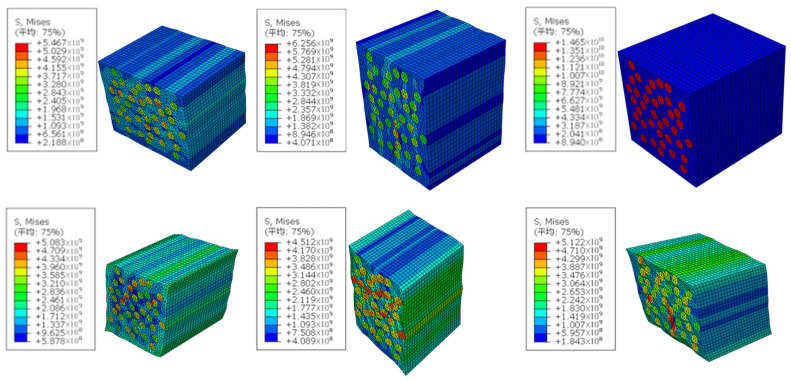
Analysis results of 6 load conditions.

The CF/PEEK unidirectional prepreg tape consists of T700 carbon fiber and a PEEK matrix, as shown in Figure 5. The size of the RVE is 50 × 50 × 50 μm, and the volume fraction of the carbon fiber is 69%. Python script is used to generate the RVE model with a random distribution of carbon fiber. The material properties of the T700 carbon fiber and PEEK are given in Table 1, and the open-source Abaqus plug-in tool EasyPBC is employed to impose periodic boundary conditions. The material properties are analyzed and calculated under six different loading conditions, as shown in Figure 6, to obtain the macroscopic homogeneous effective elastic parameters of the unidirectional prepreg tape [16,17,18].

The calculated homogeneous effective elastic parameters are assigned to the elastic properties of the unidirectional prepreg strip on the basis of the material properties. The mold temperature load, strip continuation displacement, and linear velocity are applied by adding contact constraints between the strip and coiling mold cavity to obtain stress–strain maps of the strip-coiling process. The forming load pull and the shape of the tube’s coiling cross-section are adopted as input conditions for the next process.

**Figure 7:**
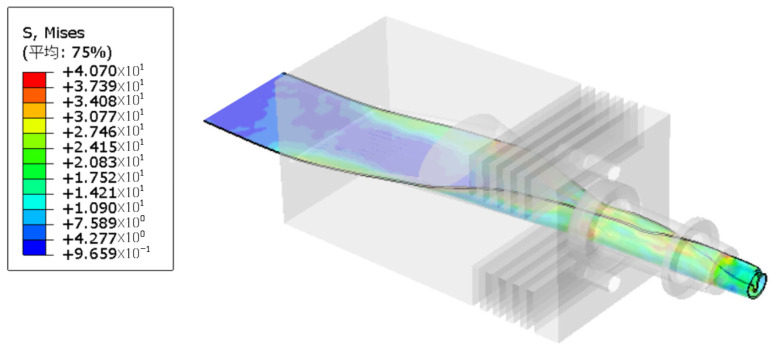
Stress cloud of strip-coiling process.

**Figure 8:**
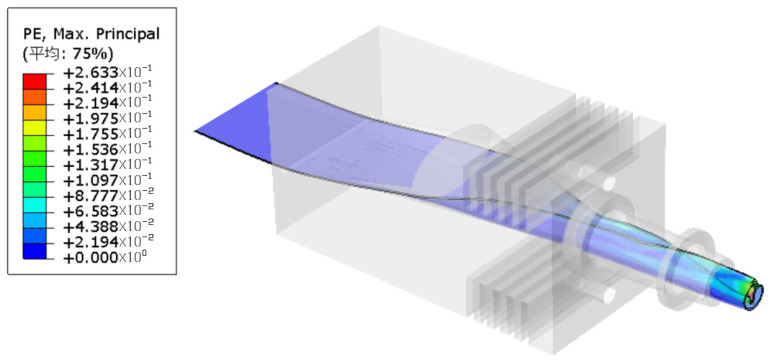
Strain cloud of strip-coiling process.

When the strip enters the mold, the stress–strain generated increases after the strip enters the precurling section and the conduit, as shown in Figure 7 and Figure 8. The load pull of the strip-curling process is generated, and the reduction in the load pull is the goal of the optimization of the mold structure.

**Figure 9:**
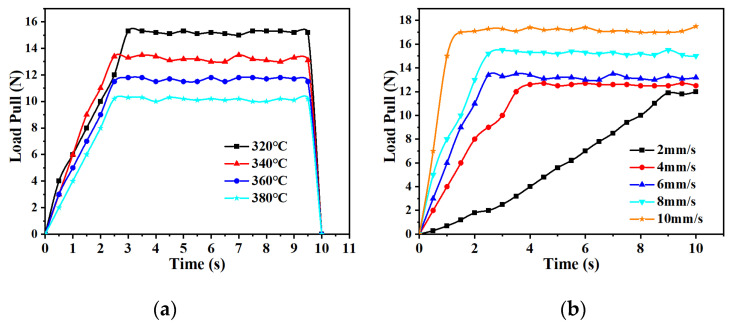
Simulation curve of forming load pull. (**a**) Under the condition of forming speed of 4 mm/s; (**b**) under the condition of forming temperature of 340 °C.

As shown in Figure 9a, the forming load pull decreases with the increase in temperature at a forming speed of 4 mm/s and increases with the increase in forming speed at a forming temperature of 340 °C, as shown in Figure 9b. The forming speed affects the heat transfer from the mold to the strip, resulting in changes in the modulus of elasticity of the base material of the CF/PEEK unidirectional prepreg strips and ultimately leading to the effect of the strip temperature on the forming load pull.

### 3.3. Hot-Melt Pultrusion of Rods

Numerical simulation of the hot-melt extrusion process of the base PEEK material is performed with COMSOL on the basis of the rolling process to form the fittings [21,22]. The simulation process assumes that PEEK does not exhibit any change in volume during melting and solidification, and the melted PEEK has a constant, uniform velocity throughout the modeling area.

When the prewound CF/PEEK strip in the form of a tube enters the hot-melt forming mold through the conduit. The base PEEK material melts under high temperature and squeezing and pulling pressures. It is in a viscous flow state and continuously flows through the mold outlet, and afterward, it cools down to a glassy state.

Under the assumption that the process is in a steady state and continuous, the heat transfer can be described by

(1) $$\rho C_{P}u\cdot \nabla T+\nabla \cdot (-k\nabla T)=Q$$

where *k*, *C_p_*, and *Q* denote thermal conductivity, specific heat, and heating power per unit volume (heat source term), respectively. ***u*** is the feeding speed of the strip prerolled into a tube. As the molten PEEK cools, it transforms from a viscous flow state to a glassy state and releases a large amount of heat. The total heat released per unit of mass is given by enthalpy change Δ*H*. Heat capacity *C_p_* varies considerably during glass transition, and the difference in specific heat before and after the transition can be approximated as

(2) $$\Delta C_{p}=\frac{\Delta H}{T}$$

Polymers containing carbon fiber reinforcement have a wide temperature transition zone, in which a mixture of solid and molten PEEK coexist in the mushy zone. The apparent heat capacity approach is used in this study to examine the heat transfer with phase transition domain conditions and to consider the sensible heat associated with glass transition. Laminar flow is considered in accordance with the following equations describing fluid velocity ***u*** and pressure *p*:

(3) $$\rho \frac{\partial u}{\partial t}+\rho u\cdot \nabla u=\nabla \cdot \left[-pI+\mu (\nabla u+{\left(\nabla u\right)}^{T})-(\frac{2\mu }{3}-\kappa )(\nabla \cdot u)I\right]+F$$

(4) $$\frac{\partial \rho }{\partial t}+\nabla \cdot (\rho u)=0$$

where *ρ* is the density (constant), *μ* denotes the viscosity, and *κ* refers to the stickiness (assumed to be zero). Source term ***F*** dampens the velocity at the interface of the modulus change, which is the transition from the viscous flow state to the glassy state. The source term is obtained from the equation [23]

(5) $$F=\frac{{\left(1-\alpha \right)}^{2}}{\alpha^{3}+\varepsilon }A_{\mathrm{mush}\;}\left(u-u_{\mathrm{cast}}\right)$$

where *α* is the volume fraction of the liquid phase, A_mush_ and ε are constants related to damping, and ***u***_cast_ is the extrusion speed of the PEEK.

The process of PEEK hot-melt extrusion is nonlinear, and an iterative approach is adopted to solve it. The locations of the transitions of the hyperplastic, viscous–fluid, and glassy states are strongly correlated with the extrusion and pulling speeds and the cooling rate, so a fine mesh is used at the critical locations of the phase transitions to solve for the changes in the material properties. The adaptive mesh refinement in this study enables the use of fine meshes around the solidification front.

**Figure 10:**
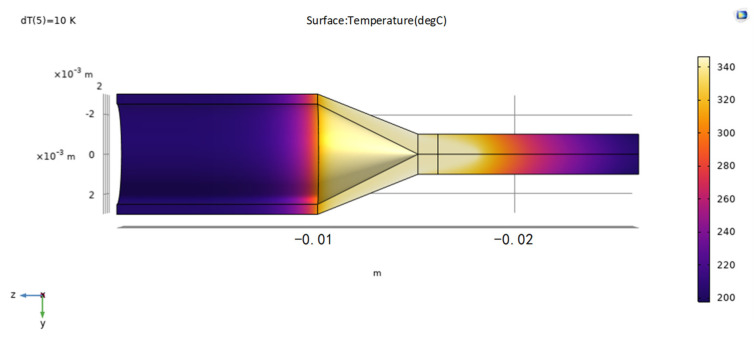
Temperature cloud diagram.

**Figure 11:**
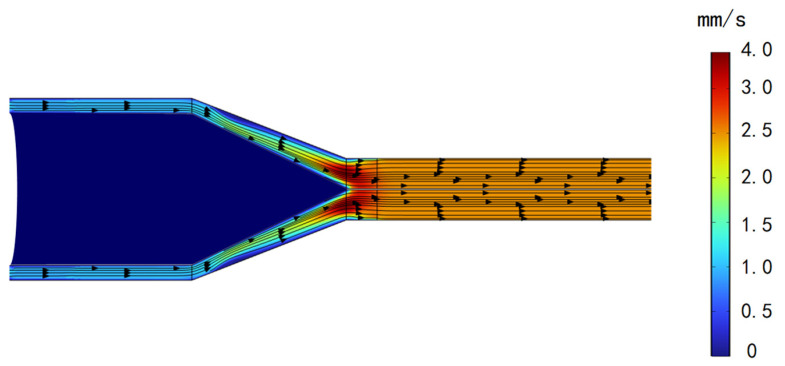
Velocity magnitude versus streamline direction.

**Figure 12:**
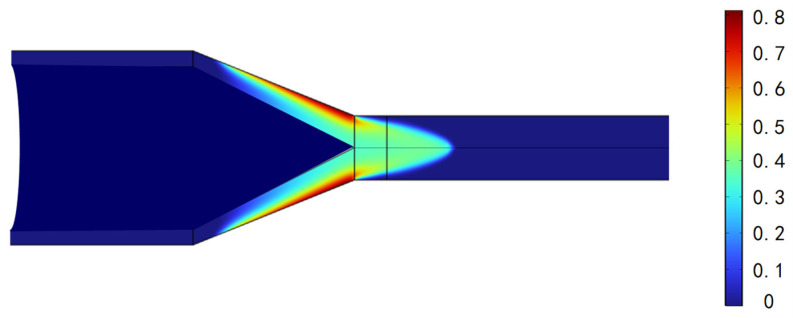
Liquid phase fraction.

The temperature distribution for the hot-melt extrusion of the tubing into rods is shown in Figure 10. Figure 11 indicates that the flow lines close to the die exit generate eddy currents, which, in a real process, results in a nonsmooth surface. The model can be used to avoid these problems and to determine the optimal mold exit design. Figure 12 shows the proportion of the distribution of PEEK that undergoes hot melting versus cool curing.

## 4. Experimental Validation and Analysis

### 4.1. Test Validation Options for Rolled Pultrusion Forming of Rods

**Figure 13:**
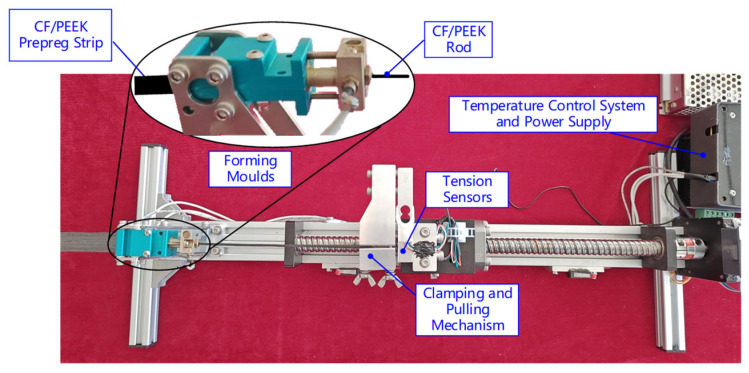
Liquid phase fraction.

In accordance with the truss structure size setting, the cross-section of the rod is 2 mm in diameter, and according to the approximate equal cross-section calculation of the rectangular cross-section of the strip and the circular cross section of the rod, the strip is 0.2 mm thick and 17 mm wide. The inlet and outlet sizes of the forming mold of the test setup are designed based on this requirement. As shown in Figure 13, the test device consists of a forming die, pull transducer, clamping and pulling mechanism, temperature control system, and power supply to achieve real-time monitoring of the forming load pull in the process of rod crimping and pultruding. The carbon fiber type of the unidirectional CF/PEEK prepreg tape is T700, and the volume fraction of the fiber is 69%.

**Figure 14:**
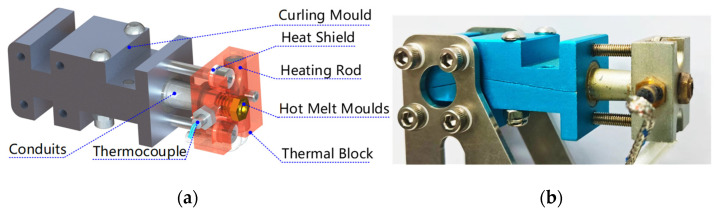
Forming mold composition and object. (**a**) Forming mold model; (**b**) physical forming mold.

The temperature gradient distribution of a single heat source is achieved through the heat dissipation and insulation design of the forming mold to complete strip curling and rod pultrusion forming. The forming mold consists of a curling mold, conduit, heat-conducting block, thermal fusion mold, heating rod, heat-insulating piece, and thermocouple, as shown in Figure 14a. The heating rod heats up the heat-conducting block through contact heat transfer, thus achieving temperature uniformity in the hot-melt mold, reducing the inertia caused by temperature fluctuations, and improving the surface quality of the rod forming. As the connecting channel between the high- and low-temperature zones, the conduit can attain a balance between heat dissipation and heat conduction through the optimized design of its structure size to achieve temperature gradient distribution in the mold.

The curling mold realizes the precurling of the strip, increases the stiffness of the strip when it is fed, and prevents wrinkling and blocking caused by friction resistance. The strip inlet is a rectangular slit, and it limits the direction of strip feeding to prevent wrinkling of the strip. The precurling section has an oval cross section, the strip is in a glassy state, and the two sides curl to the middle to form a curved strip. The hot-curl section has a circular cross-section, and the interval is a high-temperature zone. The strip transitions to a highly elastic state, and the elasticity modulus decreases, which allows the strip to be further curled to form a hollow tube. The strip is curled into a tube by the curling mold, which improves the stiffness of the strip when it is fed continuously and ensures that the rods are smoothly extruded through the hot-melt mold.

The function of the hot-melt mold is to melt the base material of the tubular strip, reorganize the carbon fiber bundles, and form a solid rod under the action of pulling and extrusion. The hot-melt mold is connected to the heat conductor block by a threaded connection, which facilitates quick replacement and repair when the mold becomes clogged and worn. The inner cavity of the mold is conical. The length of the conical surface can be shortened to prevent the adhesion of the matrix material in the viscous flow state, but the cone angle should not be so large that it prevents the carbon fiber tows from being pulled off and thus causing blockage.

### 4.2. Forming Load Pull

The forming load pulling force, as a basis for the evaluation of forming die structure design and rod forming, affects the overall energy consumption of the truss-manufacturing equipment. Energy in the on-orbit environment is valuable, so the influence mechanism of the e process parameters on the forming load pulling force is assessed to reduce the load and power consumption of the forming.

**Figure 15:**
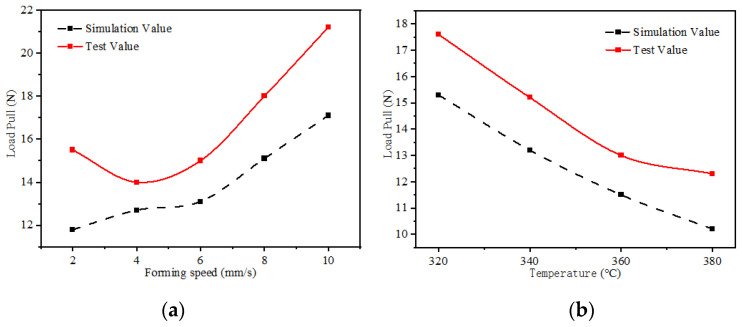
Comparison of simulated and measured values of formed load pulling force. (**a**) Under the condition of forming temperature of 340 °C; (**b**) under the condition of forming speed of 4 mm/s.

Unidirectional CF/PEEK prepreg tape with the same specification as the previously used tape is subjected to different heating temperatures and forming speeds for experimental verification. Figure 15a shows the relationship between the forming speed and load pulling force at a forming temperature of 340 °C. The trends of the simulation and measurement are different, and the measured load pulling force increases when the forming speed is too low. The reason for this is that when the speed is too low, the adhesion area of the base PEEK material becomes excessively large after melting and produces viscous friction, which requires a reduction in load to decrease the power consumption. This viscous friction also leads to increased resistance in the pultrusion process, but the simulated load does not consider the hot-melt pultrusion process. Figure 15b shows the relationship between load pull and mold temperature at the forming speed of 4 mm/s. The simulated and measured trends are the same. However, a load difference is observed between the two in the hot-melt pultrusion process of the load pull, but after 360 °C, the simulation and measurement difference is enlarged because the temperature is too high; as a result, the melting friction generated by the base material increases, resulting in increased resistance in the pultrusion process.

### 4.3. Rod Surface Quality

The surface quality of rods is used as a preliminary basis for evaluating the mechanical properties of rods. The main factors that affect the surface quality of the rods are the forming speed and temperature, so the surface of the rod in this study is scanned using a digital microscope, and the results are analyzed.

**Figure 16:**
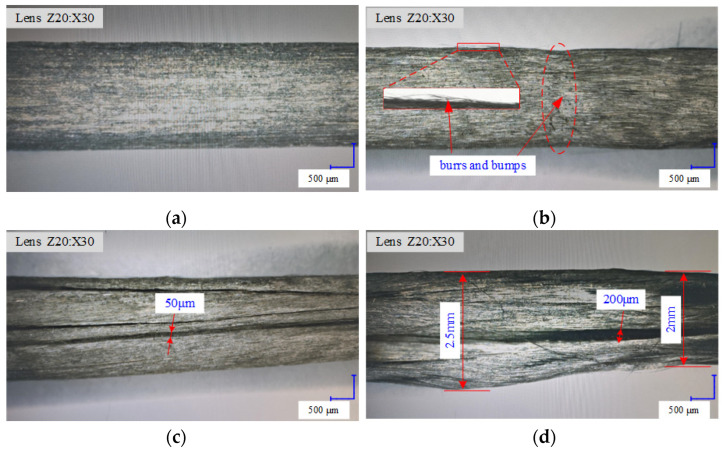
Rod surface quality. (**a**) Under the conditions of forming temperature of 340 °C and forming speed of 4 mm/s; (**b**) under the conditions of forming temperature of 340 °C and forming speed of 2 mm/s; (**c**) under the conditions of forming temperature of 380 °C and forming speed of 4 mm/s; (**d**) under the conditions of forming temperature of 340 °C and forming speed of 10 mm/s.

A comparative analysis of the surface of the rod formed under different heating temperatures and forming speed process parameters is performed. Figure 16a shows that the surface is smooth and without cracks or burrs at the forming speed of 4 mm/s and heating temperature of 340 °C. Figure 16b reveals that the surface is not smooth and has bumps and burrs when the forming speed is too low (i.e., 2 mm/s) and when the heating temperature is 340 °C. This condition leads to the melting of the adhesion of the substrate material, resulting in the uneven extrusion of the substrate material. Figure 16c,d show large cracks, but the reasons for their occurrence vary. Figure 16d reveals that when the forming speed is 10 mm/s (too high), heat transfer is reduced, the base material is not completely fused, and a crack is formed by elastic expansion after leaving the mold, which causes the diameter to increase.

### 4.4. Mechanical Properties of Rods

**Figure 17:**
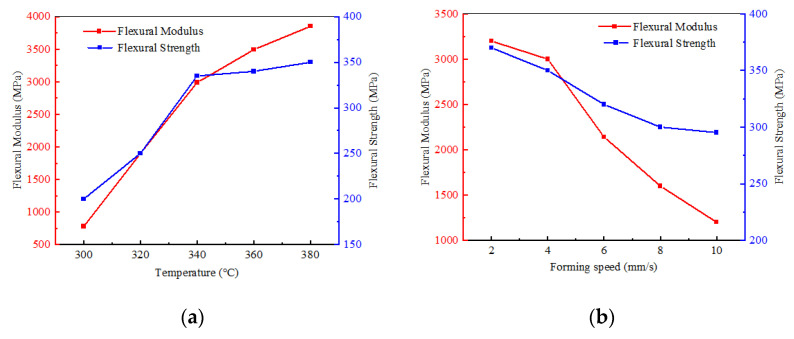
Rod bending performance trends. (**a**) Under the condition of forming speed of 4 mm/s; (**b**) under the condition of forming temperature of 340 °C.

**Figure 18:**
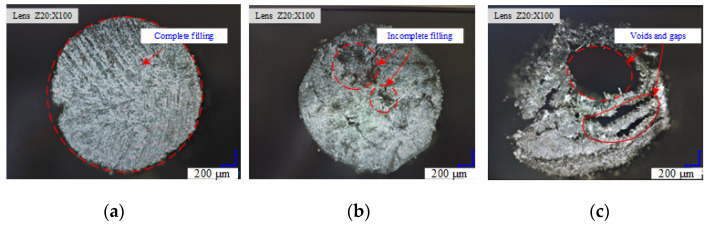
Rod section. (**a**) Under the conditions of forming temperature of 340 °C and forming speed of 4 mm/s; (**b**) under the conditions of forming temperature of 380 °C and forming speed of 4 mm/s; (**c**) under the conditions of forming temperature of 340 °C and forming speed of 10 mm/s.

The mechanical properties of rods determine the overall performance of the truss structure. In this study, the bending properties of the rods are tested, and the trend of the influence of the process parameters on these properties is analyzed to ensure that the truss structure has the optimal mechanical properties. The three-point bending properties of the bars formed under different process parameters are tested using a universal tensile testing machine. The span of the specimen is 32 mm, the diameter is 2 mm, and the loading speed is 6 mm/min. Figure 17a shows the relationship between the flexural strength and the flexural modulus of the bars with heating temperature at a forming speed of 4 mm/s. The results indicate that the mechanical properties decrease with the increase in forming speed. Figure 17b presents the relationship of the flexural strength and flexural modulus of the rods with forming speed at a heating temperature of 340 °C. With the increase in heating temperature, the mechanical properties improve, but the improvement is slow. As indicated in Figure 18, heating temperatures and forming speeds that are too high affect the density of the filling of the matrix material, which in turn influences the mechanical properties of the rods.

## 5. Conclusions

In this research, for the special environment of space, an efficient rod crimping and pultruding on-orbit isoform forming technology is developed to realize the rapid forming of raw CF/PEEK strip materials into rods. Through numerical simulation and experimental verification, the following main conclusions are derived.

(1) An efficient rod crimping and pultrusion forming technology suitable for the special environment of space is proposed, and the corresponding forming mold structure is determined. A fine RVE model of unidirectional CF/PEEK prepreg tape is established, and its macroscopic equivalent mechanical properties are obtained by applying homogenization theory. Numerical simulation of the forming process is also conducted to obtain the macroscopic homogeneous effective elastic parameters of the material.
(2) A comparative analysis of the simulation and experimental results on rod forming load pull is performed, and the effects of forming temperature and speed on the rod forming load mechanism are studied. The analytical results show that when the forming speed is fixed, the forming load pull and temperature are negatively correlated; when forming temperature is fixed, the molding speed and load pull are positively correlated. The optimal molding process parameters are determined.
(3) The influences of the surface quality and mechanical properties of the rods on the process parameters are examined through a rod-forming parameter analysis. Molding speed and molding temperature affect the surface quality of the rods. A heating temperature that is too high influences the density of the material filling, which in turn affects the mechanical properties of the rods.
(4) The optimal process parameters for a rod with a diameter of 2 mm and carbon fiber type T700 are determined. The optimal process parameters are unidirectional CF/PEEK prepreg tape with a fiber volume fraction of 69% as the raw material, a forming speed of 4 mm/s, and a forming temperature of 340 °C.

This research shows that the proposed molding technology has high efficiency, low energy consumption, and high integration. It reduces the manufacturing costs and improves manufacturing efficiency, so it can serve as a novel effective solution for the manufacturing of high-performance truss rods in the aerospace field.

## Figures and Tables

**Table 1:** Material properties of T700 carbon fiber and PEEK [19,20].

Material Name	Material Properties	Parameter Values
T700	{E1, E2}	{232, 15} GPa
	G12	15 GPa
	{υ12, υ23}	{0.2, 0.07}
	ρ	1800 kg/m^3^
PEEK	E	4 GPa
	υ	0.35
	ρ	1100 kg/m^3^

## Data Availability

Data are contained within the article.
